# Could melatonin prevent Vancomycin-induced nephrotoxicity in critically ill patients? A randomized, double-blinded controlled trial

**DOI:** 10.22088/cjim.14.1.76

**Published:** 2023

**Authors:** Saeid Abbasi, Ehsan Bigharaz, Shadi Farsaei, Marjan Mansourian

**Affiliations:** 1 Anesthesiology and Critical Care Research Center, Isfahan University of Medical Sciences, Isfahan, Iran; 2 Nosocomial Infection Research Center, Isfahan University of Medical Sciences, Isfahan, Iran; 3 Department of Anesthesiology and Critical Care, Isfahan University of Medical Sciences, Isfahan, Iran; 4 Department of Clinical Pharmacy and Pharmacy Practice, Faculty of Pharmacy, Isfahan University of Medical Sciences, Isfahan, Iran; 5 Department of Biostatistics, Isfahan University of Medical Sciences, Isfahan, Iran

**Keywords:** Melatonin, Vancomycin, Acute Kidney Injury, Intensive Care Units

## Abstract

**Background:**

Previous research showed some clinical benefits regarding the nephroprotective effect of melatonin. So, this study aimed to evaluate the beneficial effect of oral melatonin on preventing acute kidney injury (AKI) in patients who received vancomycin therapy in the intensive care unit (ICU).

**Methods:**

We performed a randomized, double-blinded, placebo-controlled pilot study in an academic hospital. Adult patients admitted to the ICU who received vancomycin with normal gastrointestinal and kidney function were randomized into treatment or placebo groups. After that, enrolled patients received a tablet of melatonin (3 mg) or placebo twice daily for seven consecutive days.

The occurrence of AKI was assessed by RIFLE criteria (by measurement of serum creatinine (SCr)) and plasma neutrophil gelatinase-associated lipocalin (NGAL) concentration. Moreover, other data related to renal functions and SOFA were also compared between groups.

**Results:**

A total of 90 patients were included in the study, while 21patients in the placebo group and 20 in the intervention group completed the study. There were no significant differences between groups regarding baseline SCr, BUN, urine output, NGAL, SOFA, and glomerular filtration rate (GFR). Our results showed that these differences remained insignificant after a 7-day follow-up between groups. However, the incidence of AKI was significantly lower in the melatonin group based on the NGAL cutoff (> 150 ng/mL).

**Conclusion:**

We detected a significant decrease in vancomycin-induced nephrotoxicity incidence in patients receiving melatonin compared to placebo. However, more clinical trials in a larger population were required to confirm this result.

Frequent use of high vancomycin doses to restrain methicillin-resistant staphylococcus aureus (MRSA) infections increased kidney injury incidence, especially in critically ill patients (1).

This adverse reaction complicated patients' medical therapy (2). However, the vancomycin-induced nephrotoxicity (VIN) is mainly mild in hospitalized patients, but critically ill patients are vulnerable to VIN, which could be associated with a higher incidence of kidney failure and mortality rate (1-3). A recent study declared that 40% of critically ill adolescent and young adult patients experienced vancomycin-associated acute kidney injury (AKI) (4).

Previous studies also introduced risk factors for VIN, such as high-dose, long-term use, high trough serum concentrations of vancomycin, and concomitant administration of nephrotoxic drugs (4-7). Preserving adequate renal perfusion and applying therapeutic drug monitoring are acceptable strategies for preventing VIN. It seems some nephroprotective agents could also prevent VIN (2, 8, 9). The exact underlying mechanism of vancomycin renal toxicity is not fully understood. However, recent research declared that proinflammatory oxidation plays a principal role in its nephrotoxicity (2). Accordingly, the effect of various antioxidants has been investigated to prevent toxicity caused by vancomycin in animal studies (8, 10).

Vitamin E, vitamin C, N-acetylcysteine, erythropoietin, and melatonin were the most frequently studied antioxidants for VIN prevention (9, 11-13). The animal study in 2005 revealed that three antioxidant compounds of Ginkgo biloba, melatonin, and alpha-lipoic acid extract have potential protective effects against VIN, which might be related to the inhibition of free oxygen radical production (10). The beneficial results of some antioxidants like vitamin E and N-acetylcysteine have been indicated for VIN prevention in previous clinical trials (9, 11). However, clinical data regarding melatonin's effect to prevent kidney toxicity of vancomycin has not been published until now.

$n=\frac{{\left(Z_{1-\frac{\alpha }{2}}+Z_{1-\beta }\right)}^{2}[P_{1}\left(1-P_{1}\right)+P_{2}(1-P_{2})]}{{\left(P_{1}{-P}_{2}\right)}^{2}}$

Safety profile and diversity of melatonin impact in central and peripheral oscillators based on its physiological regulatory effects open essential perspectives for using melatonin as clinical preventive and therapeutic applications (14). Therefore the melatonin effect as detoxification of free radicals and antioxidant actions is an exciting VIN prevention option. This study aimed to evaluate the effectiveness of melatonin supplements in preventing renal toxicity caused by vancomycin in patients receiving this antibiotic in the intensive care unit (ICU).

## Methods

The present double-blinded placebo-controlled clinical trial was conducted to investigate melatonin prophylaxis's effect on renal toxicity of vancomycin in 90 patients admitted to the Al-Zahra teaching ICU hospital affiliated to Isfahan University of Medical Sciences. The institutional review board approved this study with the ethical code number of IR.MUI.MED.REC.1395.3.861, and Iranian registry of clinical trials (IRCT) registration number of IRCT20150221021159N4. This study's inclusion criteria were all adult patients (18 years and older) who received intravenous (IV) vancomycin without administration history during the last three weeks. Patients with acute or chronic kidney disease or oral intolerance at recruitment were excluded. Moreover, those with past medication history for using other nephrotoxic or nephroprotective and antioxidant medications were not included.

We calculated a sample size of 45 patients in each group based on previous data (4), considering the significance level of 0.05 and 80% power (β= 0.8), with p1 = 0.25, p2 = 0.15 for sample size formula, to investigate the effect of melatonin tablets against placebo on preventing VIN.

We took the written informed consent of patients or their family members; after that, we randomly allocated suitable patients to melatonin or placebo groups using block randomization. We selected blocks sizes of four with a random list generated by a computer with a 1:1 concealed assignment to study groups. Placebo tablets were prepared by ordering from the pharmaceutical company manufactured melatonin tablets, in the shape and blisters precisely similar to the drug.

Eligible patients were selected conveniently from Oct 2018 until Jan 2020 and, after obtaining informed consent, they were randomly assigned to the melatonin or placebo group. Patients received 3 mg melatonin twice daily in the intervention group, while the placebo tablets were administered in the control group. All patients received standard medical and preventive support for VIN. Because renal toxicity from vancomycin usually occurs within 5 to 7 days (15), patients were followed for at least seven days and up to 14 days during hospitalization (13). The study endpoints were nephrotoxicity defined based on RIFLE criteria and changes in NGAL (neutrophil gelatinase-associated lipocalin) concentration (16, 17). The previous systematic review cutoff value > 150 ng/mL for NGAL concentration was considered to predict AKI (16). In case of renal failure, based on the treating physician's opinion, a decision was made to discontinue treatment or adjust its dose based on creatinine clearance. Blood samples were obtained at baseline and after seven days to measure NGAL concentration. By A sandwich ELISA technique. Moreover, daily serum creatinine (SCr) concentration, and urine output, were documented to determine AKI based on RIFLE criteria. Glomerular filtration rate (GFR) was also calculated based on Cockcroft-Gault Formula (18).

According to a previous systematic review and meta-analysis, the NGAL level has a diagnostic value for acute kidney injury (13). Recent studies also declared NGAL allows early diagnosis of AKI before a clinical diagnosis based on RIFLE criteria or cystatin C concentration in ICU adult patients (19, 20).  Furthermore, other studies revealed that plasma NGAL seems to be an objective and extremely sensitive biomarker for identifying AKI in infected critically ill patients (21, 22). 

Patients' demographic information (age, sex), the service of therapy in the ICU, and past medical and medication history were also recorded. In addition, data regarding vancomycin administration, including dose, rate of infusion, and duration of therapy, were collected. Statistical analysis: SPSS version 20 (IBM Corporation) software was used to perform descriptive and statistical analyses. The Kolmogorov-Smirnov test determined the normality distributions of continuous variables, and related data were described as mean±standard deviation or median (IQR1-IQR3) according to distribution. We performed the independent t-tests or Mann-Whitney U test to find differences between melatonin and placebo groups in continuous variables for parametric and nonparametric variables, respectively. In addition, Paired t-test or Wilcoxon signed-rank test was used to analyze differences in each group during the study*.* We presented categorical variables as frequency, and Chi-squared or Fisher's exact test was applied to examine any association between them and study groups. Moreover, the McNemar test was used to compare proportions of patients at or above the cutoff value of NGAL for nephrotoxicity in each group after the follow-up period. In our analyses, a p-value equal to or less than 0.05 was considered statistically significant.

## Results

**Figure 1.**
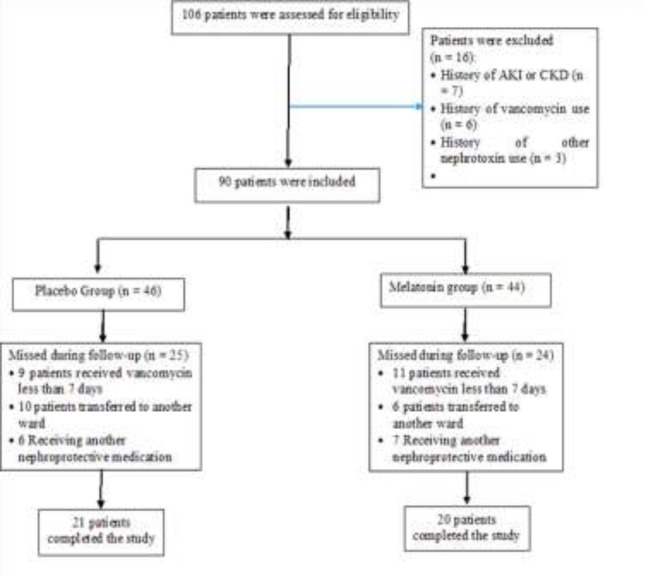
Research flowchart: how to allocate samples to two study groups

In this clinical trial, 120 patients were assessed for eligibility over one year, and after the exclusion of 16 patients, 90 patients were randomly allocated into 46 interventions and 44 controls. Finally, 20 patients in the intervention group (IG) and 21 in the control group (CG) completed the study with 14 days of follow-up. We described more explanations about the reasons for exclusion and missing follow-up in figure 1.

Vancomycin was initiated with a dose of 1 gram twice daily for all study patients and infused for 60 minutes. Baseline data of the study population are summarized in Table 1. Analysis of the data showed that there were not any significant differences in terms of demographic features and clinical or laboratory data before the intervention (P>0.05), which means homogeneity between groups. Regarding demographic characteristics, IG patients were 49.4 ±20.4 years old, and CG patients were 54.4±20.1 years old. With regard to gender, 80% were men in IG, compared to 61.9% in CG. The median SOFA score was 1, and the median Charlson score was 2 in both groups. Moreover, at baseline, the only risk factor for AKI was the concurrent receipt of nephrotoxic medications (40% and 52.4% in IG and CG, respectively). A comparative study revealed that the incidence of nephrotoxicity based on NGAL cutoff point (> 150 ng/mL) in the melatonin group (45%) was significantly lower than CG (61.9%) during vancomycin therapy (P=0.039). However, other differences between study groups about NGAL concentration, BUN, SCr, urine output, and GFR were not statistically significant. Moreover, RIFLE criteria showed that only one patient in CG experienced a renal injury while non-one was reported in IG (P<0.05). The within-group analysis also revealed that the SCr concentration decreased significantly in both groups (P=0.046 and 0.049 in IG and CG, respectively). These differences were also significant for GFR and urine output. In this regard, our result showed a substantial increase in both parameters in study groups at the end of follow-up. More details are provided in Table 2.

**Table 1:** Demographic and clinical parameters of patients at admission

Variable	Intervention Group (N = 20)	Placebo Group (N = 21)	P-Value
**Age** ^*^ **, (year)**	49.4 ± 20.4	54.4 ± 20.1	0.431
**Male sex** ^†^	16 (80)	13 (61.9)	0.203
**Service of therapy** ^†^			
**Medical**	8 (40)	5 (23.8)	0.26
**Surgical**	7 (35)	8 (38.1)	
**Trauma**	5 (25)	8 (38.1)	
**Reason of ICU admission** ^†^			
**Cardiac**	2 (10)	3 (14.3)	0.82
**Respiratory**	3 (15)	2 (9.5)	
**Complication or procedure requiring mechanical ventilation**	12 (60)	11 (52.4)	
**Complication requiring intensive hemodynamic monitoring**	3 (15)	5 (23.8)	
**Charlson comorbidity index score** **	2 (1-2.5)	2 (2-3)	0.12
**APACHE** ^*^	16.50 ± 5.09	15.71 ± 6.52	0.671
**SOFA** ^**^	1 (1 - 2)	1 (1 – 1.75)	0.81
**SBP** ^*^ **, (mmHg)**	127.6 ±14.28	129.89 ± 16.85	0.78
**DBP** ^*^ **, (mmHg)**	79.0 ± 10.01	76.10 ± 12.98	0.39
**Concurrent receipt of nephrotoxic medication** ^†^	8 (40)	11 (52.4)	0.54
**Renal function tests**			
**Creatinine** ^**^ **, mg/dL**	0.95 (0.825 – 1.1)	0.9 (0.8 – 1)	0.26
**BUN** ^**^ **, mg/dL**	14.5 (12 – 18.75)	16 (10 – 19)	0.84
**NGAL** ^**^ **, ng/mL**	489.9 (326.1 – 843.9)	442.1 (229.3 -939.8)	0.82
**GFR** ^*^ **, ml/min**	81.00 ± 25.35	80.25 ± 19.10	0.915
**Urine output** ^*^ **, mL**	2205.50 ± 591.62	2213.33 ± 811.26	0.972

* Presented as Mean ± SD and P-values are based on Independent t-Test, † Presented as Number (%) and P-values are based on Chi-square test or Exact-Fisher Test, ** Presented as Median (IQR) and P-values are based on Mann-Whitney Test. APACHE, acute physiology and chronic health evaluation; BUN, blood urea nitrogen; DBP: diastolic blood pressure; GFR: glomerular filtration rate; IQR, interquartile range; SBP, systolic blood pressure; SD, standard deviation; NGAL (neutrophil gelatinase-associated lipocalin); SOFA, sequential organ function assessment;

**Table 2:** Laboratory and clinical findings after follow up in 2 study groups

Variable	Intervention Group (N = 20)	Placebo Group (N = 21)	P-Value
**AKI incidence, n (%)**			
**Based on RIFLE criteria**	0	1 (4.8)	1^#^
**Based on NGAL concentration**	9 (45)	13 (61.9)	0.32^#^
**P-Value** ^†^	0.039	1	
**Creatinine, mg/dL, median (IQR)**	0.8 (0.7 – 1.08)	0.8 (0.75 – 0.95)	0.873*
**P-Value** ^†^	0.046	0.049	
**Difference** ^§^ **, median (IQR)**	-0.1 (-0.175 – 0)	-0.1 (-0.2 – 0)	0.96
**BUN, mg/dL, median (IQR)**	17 (13.5 – 20)	13 (9 – 17)	0.068*
**P-Value** ^†^	0.59	0.97	
**Difference** ^§^ **, median (IQR)**	0 (-3.5 – 6)	1 (-3 – 4)	0.77
**NGAL** ^**^ **, ng/mL, median (IQR)**	669.26 (370.58 – 1883.92)	680.6 (254.55 – 1039.01)	0.849*
**P-Value** ^†^	1	0.305	
**Difference** ^§^ **, mean ** ***±*** ** SD**	51.87 ± 1178.41	-353.5494 ± 2052.40	0.45
**GFR** ^*^ **, mL/min**	99.81 ± 41.10	88.14 ± 24.46	0.273*
**P-Value** ^†^	0.019	0.037	
**Difference** ^§^ **, median (IQR)**	10.3 (0 – 28.17)	10.4 (0 – 21.8)	0.565*
**Urine output** ^*^ **, mL**	2930.29 ± 517.87	3144.47 ± 1031.87	0.445*
**P-Value** ^†^	0.004	0.009	
**Difference** ^§^ **, median (IQR)**	550 (250 – 1125)	600 (45 – 1600)	0.962*
**SOFA, median (IQR)**	1 (1 – 1.75)	1 (1 – 2)	0.503*
**P-Value** ^†^	0.796	0.190	
**Difference** ^§^ **, median (IQR)**	0 (-1 – 1)	0 (0 – 1)	
**Duration of ICU stay, median (IQR)**	27 (12 – 61)	22 (12 – 51)	1*
**Duration of hospitalization, median (IQR)**	38 (14 – 61)	34 (19 – 95)	0.566*
**Mortality, n (%)**	4 (20)	6 (28.6)	0.523^#^

## Discussion

The significant decrease in the incidence of VIN by the low dose of melatonin administration declared in this study will be promising and open a new window to various indications of melatonin use in critically ill patients. However, this result was not far from the mind, and previous studies revealed the renal protective effect of melatonin in other settings in those who received nephrotoxic medications (12). The recent review declared that the beneficial effects of melatonin in different pathophysiological levels of chronic kidney disease (CKD) are related to the pleiotropic physiological actions in experimental and clinical studies (13). Another review published in 2020 suggested melatonin with comprehensive nephroprotective activity as an adjuvant to nephrotoxic drugs to improve their safety (12). 

Regarding vancomycin, it seems increased uptake in renal proximal tubular cells induced oxidative phosphorylation and generation of free radicals, which activate the apoptosis process (2). Therefore, melatonin as an antioxidant could efficiently eradicate free radical production and attenuate the endogenous antioxidant enzymes (12). So it could be concluded that melatonin has a potential effect to down-regulate the oxidative injury and cellular inflammatory process to stimulate the cellular renovation and prevention of VIN. However, no clinical study in the literature revealed the nephroprotective effect of melatonin in preventing VIN. The protective role of melatonin against VIN was only reported in a single animal study (10). In this study, Intraperitoneal administration of vancomycin 10 mg/kg for seven days caused renal toxicity, and co-administration of melatonin 10 mg/kg resulted in the restoration of renal function compared to the control group. Moreover, results showed that melatonin was more effective in restoring renal function than ginkgo biloba, α-lipoic acid, and amrinone. Therefore, this study suggested that vancomycin nephrotoxicity can be compensated with adjuvant melatonin administration (10).

As the first clinical study, our research could also represent melatonin as a safe and effective supplement to reduce VIN incidence based on the serum NGAL cutoff concentration > 150 ng/mL. However, differences in serum concentration of Cr, BUN, and NGAL and urine output and GFR before and after the intervention were not significantly different between study groups. Serum NGAL is the most sensitive marker for AKI than SCr, which lags for actual renal function assay (16). 

Although NGAL has been considered the most promising biomarker for early AKI diagnosis (23), there were some concerns due to unpredictable release from different tissues. The inability to precisely measure NGAL released by tubular cells noted in the previous study increased concerns regarding its clinical value in heterogeneous critically ill patients (24). However, various recent studies demonstrated NGAL biomarker has the best discrimination value for early detection and prediction of AKI compared to other diagnostic criteria (19, 20, 25).

Early diagnosis of AKI in critically ill and septic patients based on plasma NGAL compared to SCr or other traditional criteria may allow earlier intervention to improve patient outcomes (19, 21, 22). However, further studies with a more extensive population study could better determine the cutoff values of NGAL in diagnosing infected patients at risk of developing AKI (22). A new investigation also declared the high serum NGAL levels at ICU admission might forecast AKI development during ICU hospitalization (26).

Using the NGAL cutoff may be more appropriate than the mean difference of NGAL concentration before and after the intervention to evaluate the efficacy of melatonin to prevent VIN compared to placebo. As shown in our results, there was no significant difference between groups regarding mean differences of NGAL concentration before and after the intervention. However, the incidence of VIN based on NGAL cutoff was significantly lower in the melatonin group. 

Moreover, it should be mentioned that the SCr, GFR, and urine output were significantly ameliorated at day seven in both groups compared to the beginning. It may be related to the appropriate care in the ICU provided for all patients in the intervention and control group, such as proper hydration. The high number of missed patients during follow-up might limit our results. However, the promising results of our study will open a way to develop more clinical trials for determining the efficacy of this multipotential intervention to prevent nephrotoxicity related to vancomycin or other nephrotoxins in critically ill patients. We detected a significant decrease in VIN incidence in patients receiving melatonin compared to placebo. However, more clinical trials in a larger population were required to confirm this result. Moreover, different melatonin doses might be assessed to find the most effective amount to prevent drug-induced nephrotoxicity.
